# Indoor Particle Alpha Radioactivity Origins in Occupied Homes

**DOI:** 10.4209/aaqr.2020.01.0037

**Published:** 2020

**Authors:** Choong-Min Kang, Man Liu, Eric Garshick, Petros Koutrakis

**Affiliations:** 1 Exposure, Epidemiology, and Risk Program, Department of Environmental Health, Harvard T.H. Chan School of Public Health, Boston, MA 02215, USA; 2 Pulmonary, Allergy, Sleep, and Critical Care Medicine Section, Medical Service, VA Boston Healthcare System; Channing Division of Network Medicine, Brigham & Women’s Hospital; Harvard Medical School, Boston, MA 02130, USA

**Keywords:** Radioactivity origin, Radon, Particle radioactivity, Short-lived α-activity, Long-lived α-activity

## Abstract

Exposure to radioactivity inside homes potentially poses severe health risks which can be exacerbated by the interaction between radioactive particles and fine indoor particles; in particular, the presence of α particles are a key risk factor. Hence, in this study, particle radioactivity was concurrently measured in the family rooms and basements of 26 homes to assess its concentrations and identify its sources, both indoors and outdoors, across two seasons. The levels of radon, air ions, and particle radioactivity, which included short- and long-lived α-activity (SLA and LLA, respectively), varied greatly but were substantially higher in the basements. Also, particle radioactivity—as well as PM_2.5_ and sulfur concentrations—were lower during the heating season. SLA was associated with radon, which was consistently of indoor origin, whereas LLA was more strongly related to the sulfur measured in indoor PM_2.5_, which is a proxy of outdoor infiltration. A regression model adjusted for sulfur and SLA also indicated a predominance of outdoor sources, likely due to the short residence time of indoor particles. Our results suggest that radiation in homes originates from both the decay of indoor radon and the infiltration of outdoor radioactivity.

## INTRODUCTION

Indoor air pollution is an important contributor to the global burden of disease (WHO, 2007). In the United States, people spend over 90% of their time indoors at home, school, office, and vehicles (U.S. EPA, 1997). Indoor air may contain a large spectrum of pollutants of indoor and outdoor origin including naturally occurring radon.

Radon is a noble gas with a half-life of 3.8 days that originates from rocks and soils and tends to concentrate in enclosed spaces such as underground mines or inside houses, particularly the basement. Radon is chemically inert and is rapidly inhaled and exhaled, whereas its progeny deposits onto lung airways. Two of its short-lived progeny (^218^Po and ^214^Po) and a long-lived progeny (^210^Po) emit α radiation, which damages airway lining cells and increases cancer risk (U.S. EPA, 2003; WHO, 2009). Radon and its progeny are also the most important source of ionizing radiation in the indoor environment. Radon decays into charged metal ions. These highly reactive ions eventually attach to other existing airborne particles that can be inhaled (Offermann *et al.*, 1984; Kotrappa and Stieff, 2003). Freshly generated radon progeny initially (< 1 s) exist as unattached ultrafine clusters with diameters ranging from 0.5 to 5 nm. Within 1–100 s, these highly mobile and charged particle clusters attach to larger particles in the indoor air (Porstendorfer, 1994). When radioactive particles are inhaled, there is concern that the alpha radiation can interact with bronchial epithelial and other pulmonary cells, and potentially other tissues causing DNA damage (NRC, 1988). In many countries, indoor exposure to radioactivity is responsible for about half of all non-medical radiation exposure (UNSCEAR, 2000).

A number of survey studies have investigated residential levels of radon and its short-lived progeny (Hopke *et al.*, 1995; Darby *et al.*, 2005; WHO, 2009). These studies attributed indoor levels of radioactivity to the penetration of radon into the basement and did not take into account the indoor penetration of radioactive species from outdoor air. These radioactive species include long-lived radon progeny (^210^Pb, a half-life time of 22.3 years) that penetrates indoors, even though outdoor levels can be higher than indoor levels (Fisenne, 1993; Fisenne *et al.*, 1996). For this reason, estimating the indoor and outdoor contribution to indoor exposure to particle radioactivity may help assess and contribute to the mitigation of health risks.

In this study, we report indoor measurements of radon and particle radioactivity including short- and long-lived α-activity (SLA and LLA) from occupied homes in the metropolitan Boston area. Furthermore, we assess the indoor and outdoor origins of indoor alpha radioactivity.

## METHODS

### Sampling Scheme

We conducted indoor measurements in 26 houses of non-smokers in the Boston metropolitan area between June 28, 2017, and December 15, 2018. Each home was sampled two times for 5 days over two seasons, a non-heating and heating season. For single-family homes, we did concurrent measurements in the family room and basement. For multi-family homes, we obtained measurements in the family room where residents spend the majority of their time. Two different sampling approaches were used: simple and intensive measurements. For the simple measurements, an indoor PM_2.5_ sampler, a dual radon charcoal canister, and a CO_2_ sensor (Model AZ0003; Global Sensors, Ormond Beach, FL) were placed in the family room of 10 multi-family homes. The CO_2_ sensor with a non-dispersive infrared diffusion sensor was placed next to the indoor PM_2.5_ samplers for real-time measurements of CO_2_, temperature, and relative humidity. For the intensive measurements, we also included measurements of SLA and air ion measurements (both described below) in the family room and basement of 16 single-family homes.

### Radon and Air Ions

Radon levels were measured using passive canister samplers. The canister is 4 inches in diameter and 1 inch in thickness and contains activated carbon. In accordance with the United States Environmental Protection Agency (U.S. EPA)’s protocol, the canisters were placed at least 20 inches above the floor and 4 inches away from any indoor surface. After sample collection, the radon canisters were shipped on the same day to the Radon Testing Corporation of America (Springfield, MA) where radon levels were determined using a gamma spectrometer. The lower detection limit of this method is 3.7 Bq m^−3^. All analytical procedures were in complete accordance with current U.S. EPA protocols (EPA 402-R-92–004) for the analysis of radon in air. As per EPA recommendations for measurement reliability, duplicate radon samples were collected in most homes. Duplicate radon measurements were highly correlated with a slope of 0.88 (R^2^ = 0.86, p < 0.001). Since relative humidity at the homes was not high (mean 48%; data shown below), the effect of humidity on the measurement of radon would not expected to be significant. As another quality control, concurrent radon measurements using a charcoal sampler and an electret radon sampling unit (E-PERM; Rad Elec Inc., Frederick, MD) described in details below were highly correlated with a slope of 0.92 (R^2^ = 0.85, p < 0.001), indicating a minor effect of humidity on the charcoal method. The charcoal method was used as a routine radon measurement for this study.

For air ions, we utilized ionometers to measure positive and negative ions from only 5 single-family homes due to instrumental malfunction. An ionometer (Model IM806v2; Holbach, Germany) was used to measure unattached radon progeny (e.g., ^218^Po, ^214^Pb, ^214^Bi, and ^214^Po) expressed as the total number of positive and negative ions per cubic centimeter. The ionometer features two measurement channels for simultaneous measurement of positively and negatively charged air ions. The measuring system consists of two outer electrode tubes, each holding a central electrically insulated smaller electrode. Since the polarities of the applied voltage differ by electrode, one accelerates the positively charged ions and the other electrode the negatively charged ions to the inner electrode. Zero calibration was performed before the measurements. We performed simultaneous measurements using two ionometers which had agreement within ±5% error and the average was reported.

### Particle α-activity from the Short-lived Progeny

Short-lived α-activity attached to particles was considered a surrogate of attached short-lived radon progeny. This α-activity was measured using an electret-radon progeny integrating sampling unit (E-RPISU; Rad Elec Inc., Frederick, MD). It collects radon progeny on a 25-mm glass-fiber filter at a low-flow rate of 0.8 L min^−1^ and registers α radiation activity from the deposited progeny during the sample collection. This filter was mounted on the side of the electret ion chamber so that the collected progeny ionizes the air inside the chamber. The initially charged electret is neutralized by ions generated from radon progeny decay causing electret voltage to drop. This voltage drop is proportional to the time-integrated progeny concentration (Kotrappa and Stieff, 2003). The electret is a dielectric material carrying a permanent electrical charge and is used to monitor ions produced from α radiation emitted by radon progeny collected on a filter (Kotrappa *et al.*, 1981; Goheen *et al.*, 1994; Dua *et al.*, 1999). After sample collection, the electret voltage drop was measured with a voltage reader, which was calibrated using references provided by the manufacturer. The lower detection limit of this determination was 3.9 Bq m^−3^, and the reproducibility was 5%. In addition, the equilibrium factor (known as the *F factor*) was also calculated from the ratio of radon and SLA. The F factor is defined as the ratio of the equilibrium equivalent concentration of radon to the actual α activity concentration of radon in air (Swedjemark, 1983), and accounts for the fraction of gas-phase radon converted to particle-phase short-lived progeny.

### Particle α-activity from the Long-lived Progeny

Indoor PM_2.5_ samples were collected using a two-stage cascade impactor. The samplers collect particles in three size ranges at a flow rate of 5 L min^−1^. The two impactor stages are equipped with two slit-shaped acceleration nozzles and are followed by a backup Teflon filter at the third stage. The first two stages use polyurethane foam as impaction surfaces that collect particles above 10 μm and between 2.5 and 10 μm (Lee *et al.*, 2006; Case *et al.*, 2008). The third stage uses a 37-mm Teflon filter supported by a drain disk and a stainless-steel screen to collect PM_2.5_. Teflon filters were weighed before and after sample collection using an electronic microbalance (Model MT-5; Mettler Toledo, Columbus, OH) at a controlled temperature and relative humidity to obtain gravimetric mass concentration. Subsequently, they were analyzed for sulfur and other elements using an energy-dispersive X-ray fluorescence (EDXRF) spectrometer (Model Epsilon 5; PANalytical, The Netherlands). The lower detection limit of sulfur determination was 1.5 ng m^−3^. A detailed description of the XRF analysis is also available elsewhere (Kang *et al.*, 2014). Assuming that no significant sources of sulfur exist indoors, the indoor sulfur concentration can be used as a proxy of the infiltration of outdoor PM_2.5_ (Sarnat *et al.*, 2002).

Particle α-activity from long-lived radon progeny was measured on the PM_2.5_ filters. After storage for at least one year, α radiation was measured to evaluate particle radioactivity from long-lived progeny decay (^210^Pb to ^210^Po) using a low-background proportional counter (Model LB4200; Canberra Industries, Inc., Meriden, CT) with a P10 carrier gas (10% methane balanced with argon). We assessed various counting times and used 600 minutes to account for the low radioactivity level of the filter samples. The counter was calibrated with a 0.0518 μCi NIST traceable ^210^Po source on 5.7-cm planchet and the counting 4π efficiency was 40 ± 1%. The background level was below 0.1 counts min^−1^ and the detection limit was 0.015 mBq m^−3^ with a counting time of 600 minutes and a sample volume of 36 m^3^. All α-radiation activities except one sample measured from the filters were substantially higher than the detection limit. After one year of storage, the total α activity is dominated by ^210^Po decay, which is the progeny of ^210^Pb that has the longest half-life (22.3 years). Therefore, LLA (*C*) in the air at the time of sampling was estimated using the equation below (Sheets and Thompson, 1994):

(1) $$C\;=\;\frac{A_{\alpha \left(t\right)\cdot e^{\lambda t}}}{Q}$$

where *A*_*α*(*t*)_ is α-activity measurement (mBq); *λ* is the decay constant (8.51 × 10^−5^ d^−1^) for ^210^Pb; *Q* is air sample volume (m^3^); and *t* is the duration in days between sampling and measurement.

### Linear Mixed-effect Regression Analysis

A linear mixed-effect regression model was used to estimate the association between the measurements associated with particle radioactivity. The model is useful to analyze data that include repeated measures, such as multiple data points for each home. A random intercept was used for each home, and a coefficient was analyzed as a fixed effect.

## RESULTS

### Key Home Characteristics

In total, 26 homes that included single-family and multi-family homes participated in this study. Table 1 presents the key characteristics of homes. The single-family homes included had a ground-floor living level, and the multi-family homes consisted of 5 multi-level apartments and 5 condominiums. The year of construction ranged from 1900 to 2016 and the average living area was approximately 1,600 ft^2^. As shown in Table 1, the key home characteristics are typical of homes in the northeastern US.

### Radon and Particle Radioactivity in Family Rooms and Basements

Indoor measurements were conducted in the family room, or the family room and basement concurrently, and summarized in Tables 2 and 3. Radon levels averaged 54.9 ± 59.7 (mean ± SD) Bq m^−3^ ranging from 3.7 to 288.6 Bq m^−3^. The average radon levels in the family rooms and basements were 31.1 ± 28.3 and 105.0 ± 76.6 Bq m^−3^, respectively. Radon levels were considerably higher in basements as compared to family rooms. Radon in buildings originates from soils adjacent to the foundation, construction materials such as concrete, and tap water when supplied from radium-bearing aquifers. Note also that radon levels varied greatly across homes. The U.S. EPA recommends that residential radon levels in the basements must be below 148 Bq m^−3^, an equivalent of 4 pCi L^−1^. If the radon levels are higher than the guideline, a mitigation system is suggested to reduce levels. Among our basement radon measurements, 5 out of 18 measurements exceeded this guideline.

Positive ions in family rooms averaged 1,064 ± 636 ions cm^−3^ and negative ions averaged 981 ± 843 ions cm^−3^. In contrast, in basements, the corresponding positive ions averaged 2,876 ± 1,896 ions cm^−3^ and the negative ions averaged 2,445 ± 1,737 ions cm^−3^. The difference by a factor of about two is likely due to the higher radon levels in the basement and/or the higher air exchange in family rooms. We also found somewhat diurnal variation in positive and negative ions (Fig. S1). Positive ions were highly correlated with negative ions (R^2^ = 0.96, p < 0.001). Furthermore, radon levels were highly correlated with positive (R^2^ = 0.86, p < 0.001) and negative (R^2^ = 0.80, p < 0.001) ions, indicating that air ions are associated with radon progeny from radon decay. The unipolarity (n^+^/n^−^) of positive and negative ions was 1.4. The average air ions to radon quotients were 61 × 10^6^ ions Bq^−1^ for n^+^/Rn and 51 × 10^6^ ions Bq^−1^ for n^−^/Rn, which are slightly higher than a previous finding (40 × 10^6^ and 31 × 10^6^ ions Bq^−1^, respectively) (Kolarz *et al.*, 2009). For the basement, the association (R^2^) between positive and negative ions was 0.99 (p < 0.001) and the associations with radon were 0.90 for positive ions and 0.87 for negative ions. The unipolarity was 1.2, and the quotients of Rn/n^+^ and Rn/n^−^ were 38 × 10^6^ and 33 × 10^6^ ions Bq^−1^ for positive and negative ions, respectively. For the family room, the unipolarity was 1.5 and the quotients were 79 × 10^6^ and 65 × 10^6^ ions Bq^−1^ for positive and negative ions, respectively. Positive ions were highly correlated with negative ions (R^2^ = 0.81, p < 0.001), while air ions were poorly correlated with radon (positive ions: R^2^ = 0.29, p = 0.112; negative ions: R^2^ = 0.09, p = 0.403). The difference in associations indicates that air ions in family rooms may have originated not only from the basement radon but also from the outdoor air.

For SLA, an overall average across all home measurements was 47.4 ± 36.5 Bq m^−3^. The SLA averaged 24.9 ± 16.8 Bq m^−3^ in family rooms, while it averaged 63.9 ± 38.5 Bq m^−3^ in basements, which is over two times higher than the family room measurements. For LLA, an overall average was 1.11 ± 0.61 mBq m^−3^. The average was 1.06 ± 0.54 mBq m^−3^ in family rooms, while it was 1.25 ± 0.79 mBq m^−3^ in basements. The SLA and LLA in the basement were higher than those in the family room. As expected, the radioactivity of short-lived progeny was substantially greater than that of long-lived progeny because the decay of short-lived progeny is much faster to yield greater α-activity. Note again that two measurements represent the radioactivity derived from a different particle size: SLA represents the radioactivity of non-size-selected particles (i.e., total suspended particles), whereas LLA represents the radioactivity of PM_2.5_. However, since radioactivity is predominantly associated with fine particles due to their larger surface area (Akbulut *et al.*, 2012), the radioactive measurements from different particle sizes would not meaningfully contribute to the difference.

The exposure to indoor PM_2.5_ is important not only for toxicity due to trace metals but due to attached particle radioactivity (i.e., SLA and LLA) because it can be deeply inhaled. Indoor sulfur concentrations of PM_2.5_ were used to determine the infiltration of outdoor PM_2.5_. Outdoor PM_2.5_ can enter the home through windows, doors, cracks and other openings in the structure, and as make-up air in heating and air-conditioning systems. Overall PM_2.5_ and sulfur concentrations across all home measurements were 6.0 ± 3.7 and 0.189 ± 0.109 μg m^−3^, respectively. The PM_2.5_ concentrations averaged 7.0 ± 3.9 μg m^−3^ and sulfur concentrations averaged 0.193 ± 0.117 μg m^−3^ in family rooms. In comparison, basement concentrations were lower with PM_2.5_ averaging 3.6 ± 2.0 μg m^−3^ and sulfur averaging 0.153 ± 0.084 μg m^−3^. This difference can be explained by the greater infiltration of outdoor PM_2.5_ and sulfur into the living area compared to the basement.

### Radon and Particle Radioactivity by Season

**Fig. 1.**
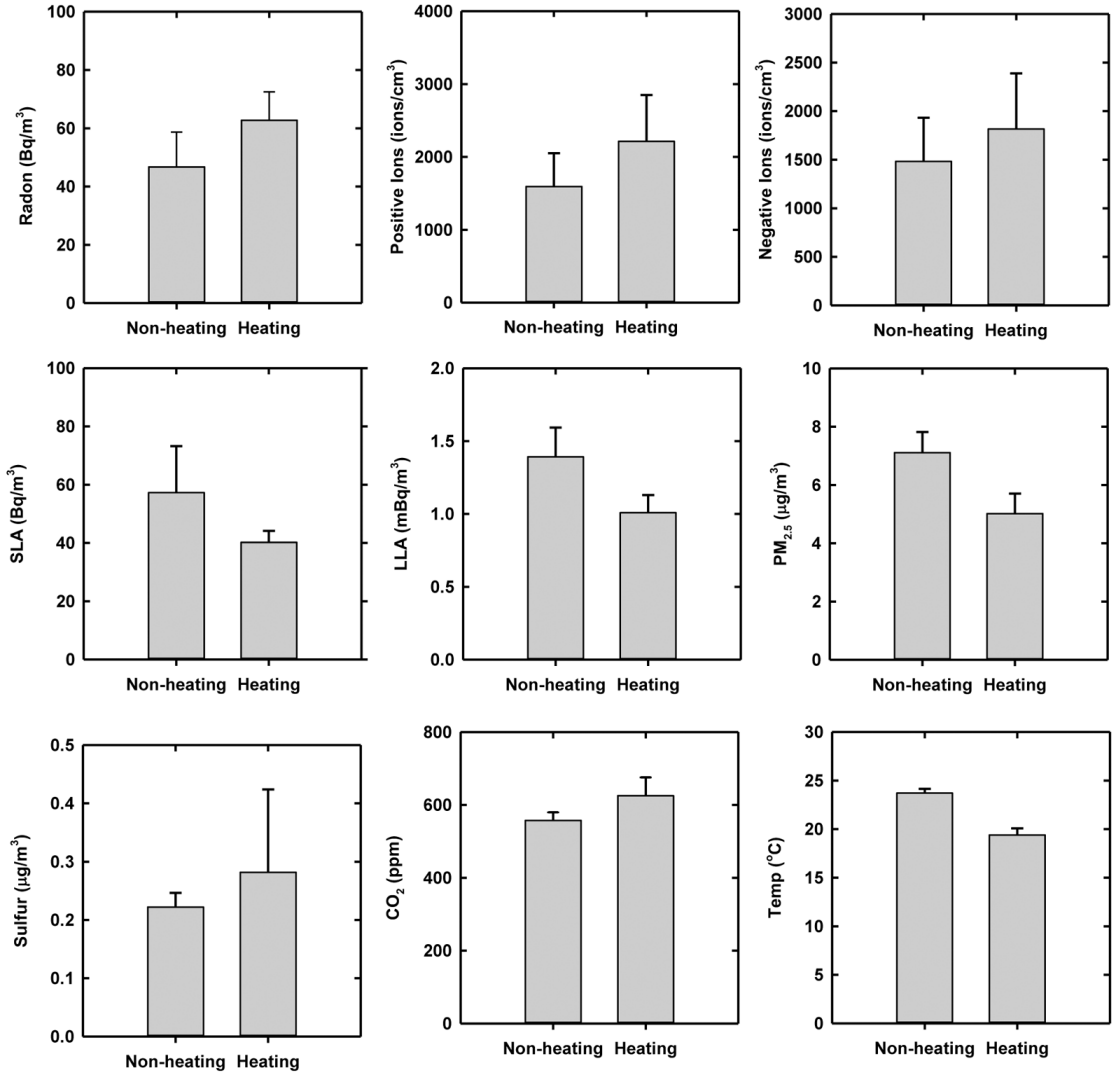
Radon and particle radioactivity for non-heating and heating seasons.

Indoor measurements were also conducted for non-heating (June–September) and heating (October–March) seasons in the same homes. Fig. 1 shows the average and standard error of seasonal measurements. The summaries are also available in Table S1 (Appendix). Radon averaged 48.0 ± 67.3 Bq m^−3^ for the non-heating season, while it averaged 62.8 ± 49.6 Bq m^−3^ for the heating season. Positive and negative ions for non-heating season averaged 1,595 ± 1,447 and 1,484 ± 1,420 ions cm^−3^, respectively. As for the seasonal variation of radon, air ions were greater for the heating season, with averages of 2,213 ± 1,807 positive ions cm^−3^ and 1,817 ± 1,621 negative ions cm^−3^. The same variation in radon and air ions by seasons and places indicates their relevance, as found in previous studies (Kolarz *et al.*, 2009). SLA and LLA for non-heating season averaged 57.3 ± 53.0 Bq m^−3^ and 1.22 ± 0.57 mBq m^−3^, respectively, whereas they averaged 40.2 ± 15.3 Bq m^−3^ and 1.01 ± 0.64 mBq m^−3^, respectively, for the heating season. The PM_2.5_ and sulfur concentrations for non-heating season averaged 7.1 ± 3.6 and 0.231 ± 0.120 μg m^−3^, respectively, whereas they averaged 5.0 ± 3.6 and 0.137 ± 0.076 μg m^−3^, respectively, for the heating season. The PM_2.5_, sulfur, SLA, and LLA were much higher for the non-heating season than the heating season, which is the same variation of seasonal PM_2.5_ and sulfur concentrations measured at the Harvard Supersite located at downtown Boston (Kang *et al.*, 2010).

### Regression Analysis Results

**Fig. 2.**
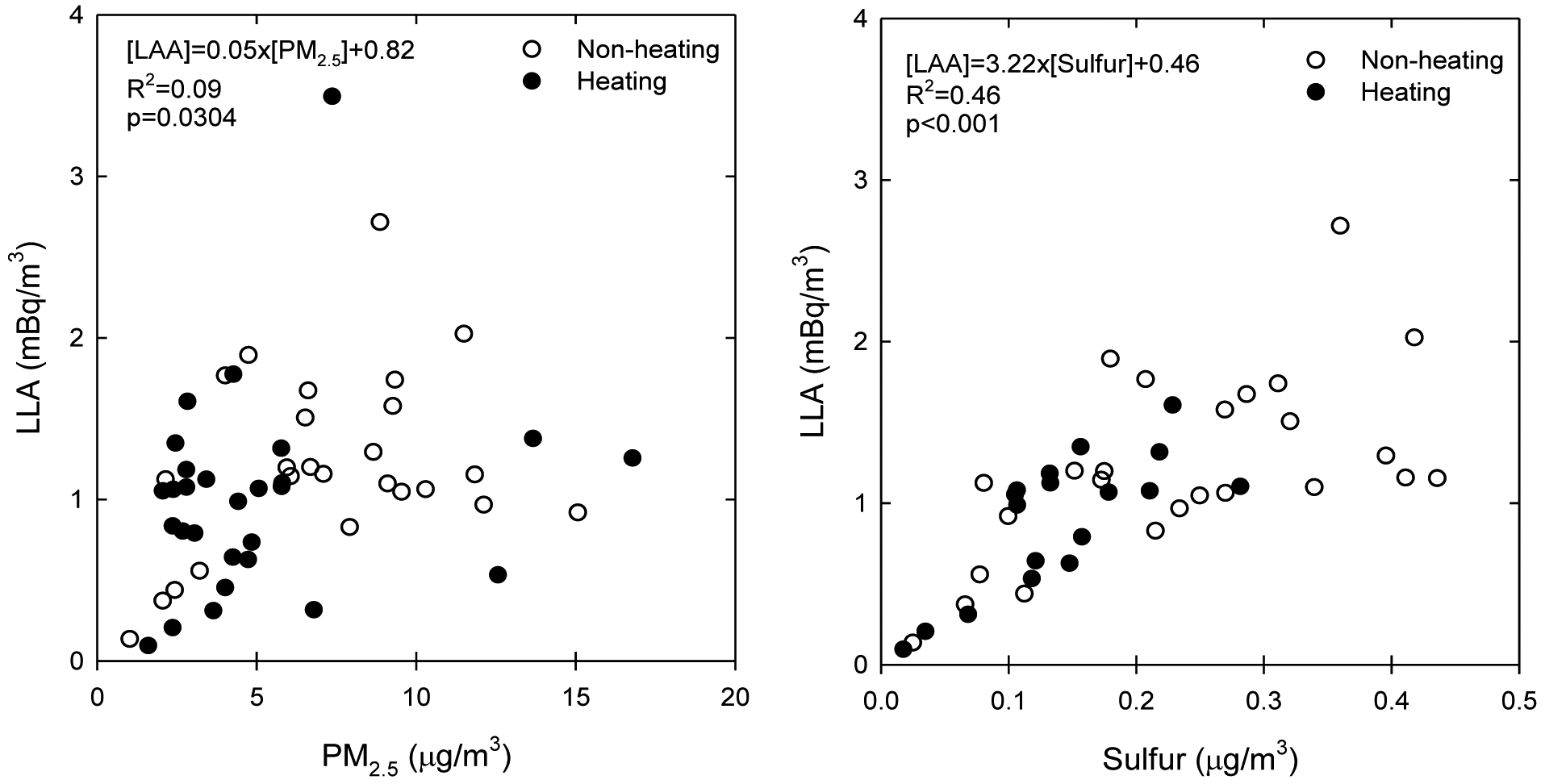
Associations between long-lived α-activity (LLA), PM2.5 and sulfur concentrations.

Table 4 summarizes the univariate associations between the species associated with particle radioactivity, PM_2.5_, and sulfur concentrations. Linear mixed-effect regression indicated that SLA was significantly associated with radon (R^2^ = 0.55, p < 0.001), and LLA was significantly associated with sulfur (R^2^ = 0.46, p < 0.001). Fig. 2 also compares the associations of LLA with PM_2.5_ and sulfur. LLA was associated more with sulfur (R^2^ = 0.46, p < 0.001) than PM_2.5_ (R^2^ = 0.09, p = 0.030) in all seasons. Sulfur is likely a better proxy of outdoor infiltration than PM_2.5_, with no seasonal variation. This may be because indoor PM_2.5_ could also be affected by indoor PM_2.5_ generated from cooking, gas stoves or kerosene heaters (Dockery and Pope, 1981; Derbez *et al.*, 2018). In contrast, the radon was not significantly associated with all other variables except SLA. The SLA was not significantly associated with LLA, PM_2.5_, and sulfur, whereas the LLA was not significantly associated with radon, SLA, and PM_2.5_.

## DISCUSSION

Radon progeny attached to existing particles (especially PM_2.5_) are of great concern as the radioactive particles can be inhaled and deposited in lung and subsequently translocate to other organs. This risk results in chronic exposure given the long lifetime of the progeny (e.g., ^210^Pb: half-life time = 22.3 years) and if strategies to vent enclosed spaces regularly and/or to limit the ingress to such places are not adopted. In this study, we characterized indoor exposure to radon and particle radioactivity by measuring air ions, SLA and LLA. Furthermore, we estimated the indoor and outdoor origins of radon progeny that contribute to indoor particle radioactivity.

Overall radon levels were estimated to be 54.9 Bq m^−3^ ranging from 3.7 to 288.6 Bq m^−3^ in the study area: The radon levels in basements (105.0 Bq m^−3^) were over three times higher than in family rooms (31.1 Bq m^−3^). The Iowa radon lung cancer study including 1,027 home measurements observed that radon levels were lower at higher floors with averages of 170, 89 and 70 Bq m^−3^ for the basement, first and second floors, respectively (Field *et al.*, 2000). Thirteen European radon-cancer studies (Darby *et al.*, 2005) found a mean radon level of 97 Bq m^−3^ in family rooms. Dudney *et al.* (1990) consistently found a higher level in basements than in living areas both during the summer (26–420 Bq m^−3^ in living areas vs. 53–530 Bq m^−3^ in basements) and the winter (8–370 Bq m^−3^ in living areas vs. 17–650 Bq m^−3^ in basements) in 70 homes of the southern U.S. Compared to these studies, our radon levels are slightly lower for several reasons such as inclusion of multi-floor apartments, better ventilation, different geology, and different construction materials (more wood, less mineral). Besides, based upon the EPA classification Iowa is in a higher-radon area (Zone 1, highest-radon-potential area) than Boston (Zone 2, moderate-radon-potential area). Air ions (positive and negative ions) were highly correlated with radon levels, suggesting that air ions are associated with radon decay. However, the association in family rooms only was weak possibly due to the intrusion of outdoor air ions. Contrary to a consistent diurnal variation in a multi-level unoccupied new building (Kolarz *et al.*, 2009), we did not find the significant diurnal pattern in the levels of air ions. This may be because each home has radon sources (e.g., basement), which may be associated with a variety of potential ventilation mechanisms.

The equilibrium factor was calculated from a ratio of radon and SLA. Estimating the factor is important in risk assessment related to inhalation of radon. Our estimated factors in family rooms and basements were 0.59 and 0.59, respectively. Swedjemark (1983) found that the F factors ranged from 0.28 to 0.80 and were influenced by multiple factors including house types, locations, and air exchange rates. Hopke *et al.* (1995) estimated an equilibrium factor of 0.41 from 7 home measurements of radon and its progeny in the northeastern U.S. and southeastern Canada. The U.S. EPA also assumed a factor of 0.5 to establish the action level of 148 Bq m^−3^ for home remediation (Bruno, 1983). Our findings on the equilibrium factors are reasonable and within the reported range by previous studies.

The LLA in basements was higher than that in family rooms, suggesting that the LLA might partly be affected by indoor SLA even though the non-significant association was found from the regression analysis. Similar to the LLA, the SLA was also higher in basements. Dudney *et al.* (1990) found that the short-lived radon progeny in family rooms were approximately half of the basement measurements in the southern U.S. cities. Fisenne *et al.* (Fisenne, 1993; Fisenne *et al.*, 1996) determined the LLA on archived indoor air filters collected in a twelfth-floor apartment in southern New Jersey (0.34 mBq m^−3^ ranged from 0.08 to 0.82 mBq m^−3^) and in a fifth-floor office in New York City (0.15 mBq m^−3^ ranged from 0.07 to 0.40 mBq m^−3^). In addition, they reported outdoor LLA levels were up to four times higher than corresponding indoor ones. For our study, even though we did not measure outdoor radioactivity, the outdoor LLA levels might also be higher than indoor ones in Boston. The higher activity in the non-heating season is likely because more particle radioactivity can come from outdoors, especially during this open-window season. Furthermore, the levels of LLA in our study were higher than those observed in New Jersey and New York City, possibly due to the higher radon potential in the Boston metropolitan area. The PM_2.5_ and sulfur concentrations in family rooms were higher than those in basements, whereas the LLA was higher in basements. However, in common, they were significantly higher during the non-heating season than the heating season. This can also be explained by greater air ventilation in the living area than in the basement. The summer is a preferable season to open windows rather than the winter. As discussed above, we used sulfur as a proxy of outdoor PM_2.5_ infiltration. This could be confirmed by the higher sulfur concentrations in family rooms and for the non-heating season. Overall, the PM_2.5_ and sulfur concentrations in this study were similar to the previous results (8.8 and 0.30 μg m^−3^ for PM_2.5_ and sulfur, respectively) in the metropolitan Boston region (Tang *et al.*, 2018).

Radon levels in this study exhibited significant seasonal variation as previous studies did. The seasonal variability was likely associated with the changes in the air exchange rate (e.g., frequency and duration of opening doors or windows) and radon entry (i.e., outdoor-indoor temperature gradient and the resulting soil-indoor pressure difference) by seasons. Several studies have also demonstrated a significant seasonal variation in indoor radon levels with a higher level in the winter than in the summer (Neville and Hultquist, 2008; Miles *et al.*, 2012; Giagias *et al.*, 2015). A radon survey in Athens, Greece, demonstrated that in the summer, indoor radon ranged from 42 to 186 Bq m^−3^ with 36% of residences exceeding the WHO guideline level of 100 Bq m^−3^. In contrast, in the winter, the levels ranged from 79 to 245 Bq m^−3^ with 60% of residences exceeding the guideline (Giagias *et al.*, 2015). Particle radioactivity, SLA and LLA exhibited a similar variation with a higher value for the non-heating season, whereas radon and air ion levels exhibited a similar variation with a higher value for the heating season. Similar to our findings, Dudney *et al.* (1990) found that in the southern U.S. cities, the short-lived radon progeny in living areas ranged 22–356 Bq m^−3^ in the summer and 13–463 Bq m^−3^ in the winter. Airborne particle concentrations either indoors or outdoors are thought to play an important role in determining the fate of newly generated radon progeny. Newly generated progeny from radon decay can undergo one of three processes: They can attach to existing airborne particles, a wall or other surface, or undergo radioactive decay before either of the first events occurs (NRC, 1988; Dudney *et al.*, 1990). In this study, we found an association between radon and SLA with an R^2^ of 0.55 (p < 0.001). In contrast, we also found a different seasonal variation in radon and SLA. This discrepancy may be explained by a greater equilibrium factor (along with a higher PM_2.5_ concentration) for the non-heating season, which may also indicate that PM_2.5_ levels can be a factor to determine the SLA levels from radon decay.

**Fig. 3.**
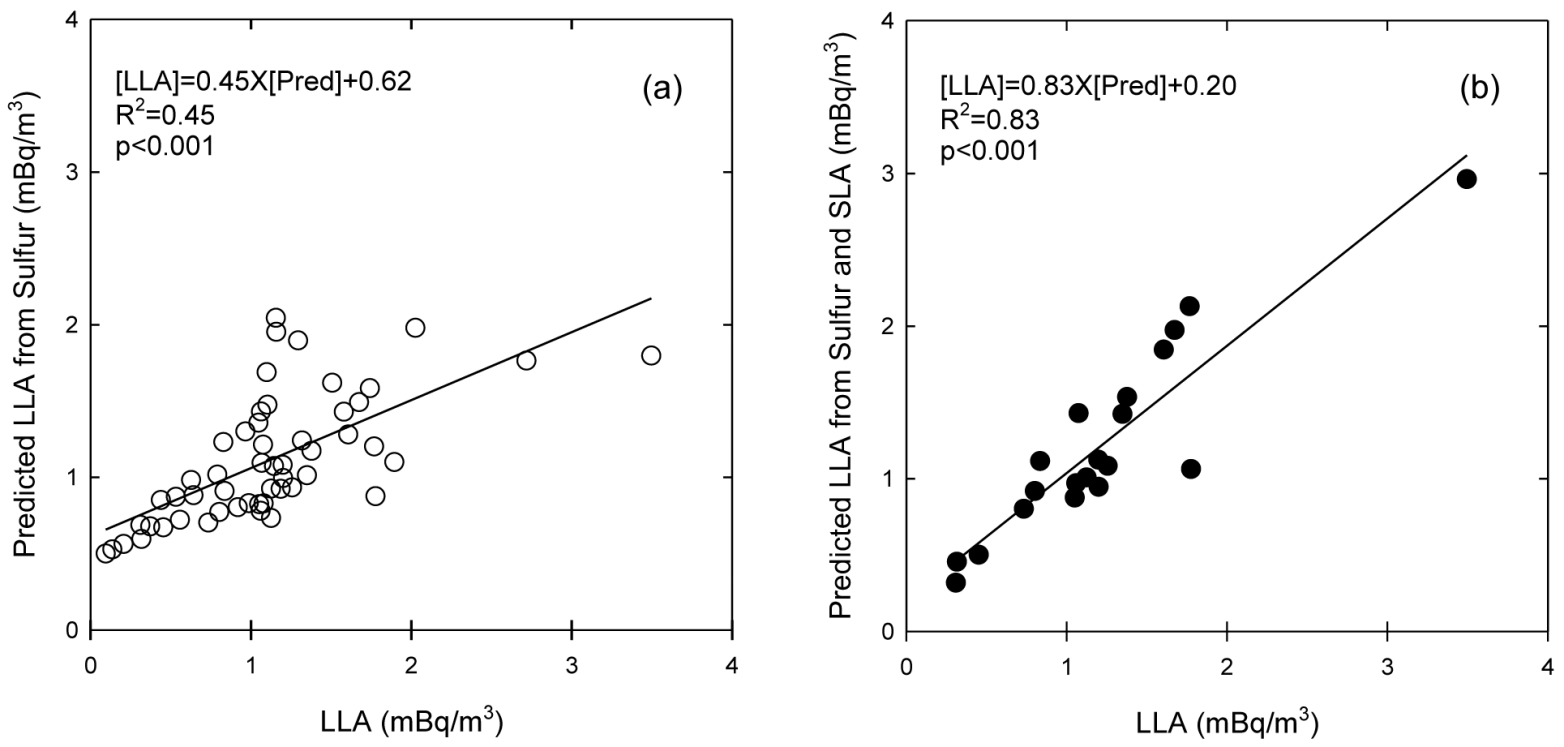
Associations between the measured LLA and the predicted LLA from SLA and sulfur concentrations.

We did not find a significant association between radon and LLA. Considering the short residence time of indoor particles due to high infiltration in typical U.S. homes, indoor LLA may mainly be associated with the outdoor radioactivity. This is also supported by the higher outdoor levels than indoor levels in previous findings (Fisenne *et al.*, 1996). However, the outdoor radioactivity might not be the only variable to account for the observed LLA. A higher LLA in basements was observed compared to family rooms. This may indicate some SLA can convert to LLA on the particles in stagnant air space such as the basement. To confirm this, a mixed regression model was used to obtain a coefficient and intercept between the observed LLA and either sulfur only (a proxy of outdoor radioactivity) or sulfur and SLA (a proxy of indoor radioactivity). The predicted values calculated from these model outputs were compared with observed LLA in Figs. 3(a) and 3(b). Compared to the regression model in Fig. 3(a) which was built with sulfur, the multivariate regression model built with sulfur and SLA was a better predictor of indoor LLA, with a higher slope and a lower intercept (Fig. 3(b)). This may indicate that indoor LLA was likely associated with mainly outdoor radioactivity, as well as partially indoor radon decay.

## CONCLUSIONS

The levels of radon, particle radioactivity (SLA and LLA), and PM_2.5_ were concurrently measured in occupied homes. Indoor exposure to radon and α particles, which commonly occurs in residences, warrants great concern because of its potential health risks, which are increased by the presence of fine particles, as this fraction can be deeply inhaled. We found that the radon concentration and the particle radioactivity varied greatly among the homes and also differed by room (family room vs. basement) and season (heating vs. non-heating period). Additionally, we investigated sources of particle radioactivity and discovered that SLA was primarily associated with indoor radon decay, whereas LLA was likely connected to outdoor radioactivity. Thus, our findings suggest that both the outdoor particle radioactivity and the indoor radon progeny concentration must be considered when assessing radiation exposure indoors; toward this objective, archived air filter analysis may be useful in estimating the radioactivity inside and outside homes. Finally, we note that our sample size limits our ability to evaluate correlations, thus decreasing the statistical power and generalizability of our results.

## Supplementary Material

Supplement

## Figures and Tables

**Table 1.** Key home characteristics.

Characteristic	Number of homes
*House type:*	
Single-family	16
Multi-family	10
*Heating type:*	
Forced air	8
Radiator	18
*Heating fuel:*	
LNG^1^	15
Oil	11
*Cooking fuel:*	
LNG^1^	10
Electricity	16
*Central AC*^2^:	
Yes	12
No	14
*Ventilation:*	
Exhaust/supply	8
Natural	18

1 Liquid natural gas.

2 Air conditioner.

**Table 2.** Overall data summary.

Species	Mean	SD	Min	Max	N
Radon (Bq m^−3^)	54.9	59.7	3.7	288.6	56
Positive ions (ions cm^−3^)	1,870	1,598	490	6,240	18
Negative ions (ions cm^−3^)	1,632	1,476	132	5,403	18
SLA^1^ (Bq m^−3^)	47.4	36.5	5.3	164.4	26
LLA^2^ (mBq m^−3^)	1.11	0.61	0.09	3.50	54
F factor^3^	0.59	0.21	0.23	0.92	18
PM2.5 (μg m^−3^)	6.0	3.7	1.0	16.8	54
Sulfur (μg m^−3^)	0.182	0.109	0.018	0.436	54
CO_2_ (ppm)	592	191	399	1,410	49
Temp (°C)	21.6	3.6	12.7	27.1	50
RH(%)	48	15	14	87	50

1 Short-lived α-activity.

2 Long-lived α-activity.

3 Equilibrium factor; because this factor requires concurrent measurements of radon and SLA, the data size is lower than other variables.

**Table 3.** Radon and particle radioactivity in family rooms and basements.

Species	Family room					Basement				
	Mean	SD	Min	Max	N	Mean	SD	Min	Max	N
Radon (Bq m^−3^)	31.1	28.3	3.7	96.2	38	105.0	76.6	11.1	288.6	18
Positive ions (ions cm^−3^)	1,064	636	490	2,231	10	2,876	1,896	928	6,240	8
Negative ions (ions cm^−3^)	981	843	132	3,036	10	2,445	1,737	881	5,403	8
SLA^1^ (Bq m^−3^)	24.9	16.8	5.3	52.4	11	63.9	38.5	13.5	164.5	15
LLA^2^ (mBq m^−3^)	1.06	0.54	0.09	2.72	39	1.25	0.79	0.14	3.50	14
F factor^3^	0.59	0.09	0.45	0.69	5	0.59	0.24	0.23	0.92	13
PM2.5 (μg m^−3^)	7.0	3.9	1.6	16.8	39	3.6	2.0	1.0	7.9	15
Sulfur (μg m^−3^)	0.193	0.117	0.018	0.436	39	0.153	0.084	0.025	0.369	15
CO_2_ (ppm)	596	160	432	1,135	36	580	265	399	1,410	13
Temp (°C)	22.2	3.3	12.9	27.1	36	19.9	4.0	12.7	25.0	14
RH (%)	48	14	14	69	36	46	18	16	87	14

1 Short-lived α-activity.

2 Long-lived α-activity.

3 Equilibrium factor.

**Table 4.** Univariate regression results.

Dependent variables	Radon		SLA		LLA	
	Coefficient	*p*-value	Coefficient	*p*-value	Coefficient	*p*-value
Radon	-	-	0.403	< 0.001	0.003	0.102
SLA^1^	1.358	< 0.001	-	-	0.009	0.231
LLA^2^	18.22	0.102	9.209	0.231	-	-
PM_2.5_	−4.513	0.031	−1.865	0.298	0.048	0.030
Sulfur	−147.9	0.043	−15.29	0.859	3.696	< 0.001
F factor	−159.5	0.039	25.12	0.580	−0.984	0.367

1 Short-lived α-activity.

2 Long-lived α-activity.

3 Equilibrium factor.
