# Supplementary material for: Indoor Particle Alpha Radioactivity Origins in Occupied Homes
Source: Aerosol Air Qual Res. Author manuscript; Available in PMC 2021 Jan 1. (PMC7425685; doi:10.4209/aaqr.2020.01.0037)
Supplement: Supplement [file NIHMS1592932-supplement-Supplement.docx]

Supplementary

Table S1. Radon and particle radioactivity for non-heating and heating seasons.

| Species | Non-heating | | | | | Heating | | | | |
| --- | --- | --- | --- | --- | --- | --- | --- | --- | --- | --- |
|  | Mean | SD | Min | Max | N | Mean | SD | Min | Max | N |
| Radon (Bq/m^3^) | 48.0 | 67.3 | 3.7 | 288.6 | 30 | 62.8 | 49.6 | 5.6 | 199.8 | 26 |
| Positive Ions (ions/cm^3^) | 1,595 | 1,447 | 490 | 5,160 | 10 | 2,213 | 1,807 | 552 | 6,240 | 8 |
| Negative ions (ions/cm^3^) | 1,484 | 1,420 | 132 | 4,720 | 10 | 1,817 | 1,621 | 271 | 5,403 | 8 |
| SLA (Bq/m^3^) | 57.3 | 53.0 | 7.7 | 164.4 | 11 | 40.2 | 15.3 | 5.3 | 56.4 | 15 |
| LLA (mBq/m^3^) | 1.22 | 0.57 | 0.14 | 2.72 | 25 | 1.01 | 0.64 | 0.09 | 3.50 | 28 |
| F factor | 0.63 | 0.18 | 0.44 | 0.89 | 7 | 0.56 | 0.23 | 0.23 | 0.92 | 11 |
| PM_2.5_ (µg/m^3^) | 7.1 | 3.6 | 1.0 | 15.1 | 26 | 5.0 | 3.6 | 1.6 | 16.8 | 28 |
| Sulfur (µg/m^3^) | 0.231 | 0.120 | 0.025 | 0.436 | 26 | 0.137 | 0.076 | 0.018 | 0.369 | 28 |
| CO_2_ (ppm) | 560 | 114 | 432 | 839 | 25 | 625 | 246 | 399 | 1410 | 24 |
| Temp (ºC) | 23.7 | 2.2 | 19.3 | 27.1 | 25 | 19.4 | 3.4 | 12.7 | 25.0 | 25 |
| RH (%) | 58 | 8 | 45 | 87 | 25 | 37 | 13 | 14 | 60 | 25 |


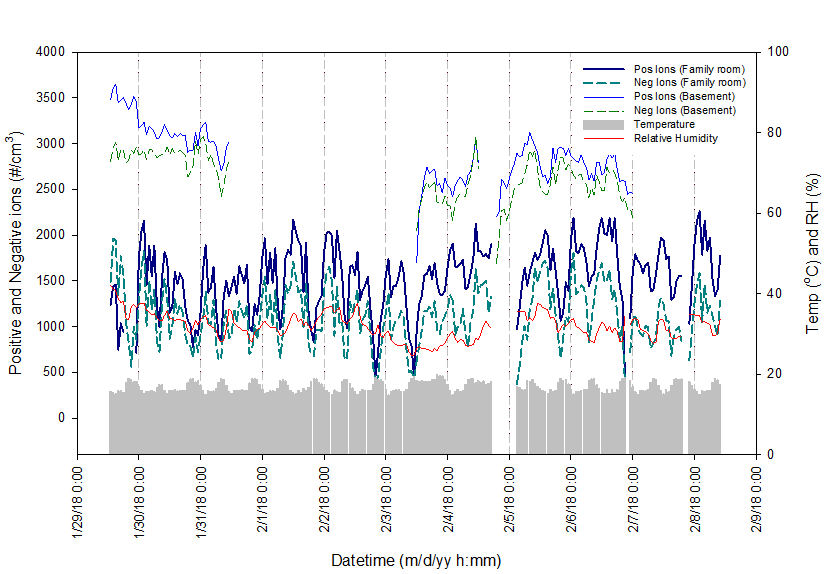


**Fig. S1.** Concurrent measurements of positive and negative ions in the family room and basement of a home during the winter; Temperature and relative humidity were measured in the family room; due to instumental malfunction of auto calibration mode the data recording was not succes under a higher level measurement; We found a high correlation between relatve humidity and radon levels at this home, but note that all home measurements did not represent this realtionship.
